# Polymodal Activation and Desensitization of TRPV1 Receptor in Human Odontoblasts-Like Cells with Eugenol

**DOI:** 10.1155/2020/8813979

**Published:** 2020-12-29

**Authors:** Karen L. Latorre, Paula A. Baldion

**Affiliations:** Grupo de Investigaciones Básicas y Aplicadas en Odontología (IBAPO), Universidad Nacional de Colombia, Bogotá, Colombia

## Abstract

Dentinal hypersensitivity is a frequent reason for dental consultation, and its pathophysiology has not been fully clarified. Previous findings have made it possible to establish a relationship between the cellular sensory capacity and the activation of the polymodal transient receptor potential vanilloid 1 (TRPV1), which is responsible for the nociceptive response and whose desensitization could cause analgesia. Thus, the objective of this study was to determine the expression, localization, and functional activity of TRPV1 in human odontoblasts-like-cells (hOLCs) and the effect of eugenol (EUG) on its activation and desensitization. Human dental pulp stem cells (hDPSCs) were obtained from third molars and were characterized using flow cytometry, and their differentiation potential toward the osteoblastic, chondrogenic, and adipogenic lineages was investigated. Subsequently, the hDPSCs underwent odontogenic differentiation for 7, 14, and 21 days, and their phenotype (odontogenic markers dentin matrix protein-1 (DMP-1) and dentin sialoprotein (DSP)) was evaluated using immunofluorescence. The TRPV1 gene expression in hOLCs was estimated using RT-qPCR, and its localization was analyzed using immunofluorescence. Half-maximal effective concentration (EC50) from both eugenol (EUG) and capsaicin (CAP) was determined; in addition, receptor activation was evaluated against chemical, thermal, and pH stimuli. For the statistical analysis, a one-way ANOVA with a Tukey post hoc test (*p* < 0.05) was used. After establishing the *in vitro* model of hOLCs and the membrane location of TRPV1, its chemical activation with EUG and CAP was demonstrated, as well as its thermal activation at ≥ 43°C and with an acidic (<6) or basic pH (between 9 and 12). Receptor desensitization was achieved after 20 min of exposure to two concentrations of EUG (603.5 and 1000 *µ*M). These findings represent a stepping-stone for the construction of a pulp pain study model oriented toward a therapeutic alternative for the treatment of dentinal hypersensitivity.

## 1. Introduction

Dentinal hypersensitivity continues to be a frequent reason for dental consultations. It occurs as a short, transitory, and acute pain that affects 8–57% of the adult population. Despite its high prevalence, the exact cause of this exacerbated nociceptive response to external stimuli, whether chemical, thermal, or mechanical, is not yet known [1]. Three main theories have been postulated to explain this physiopathogenesis: the first, named “Nerve theory,” explains sensitivity as a result of direct stimulation of the afferent nerve fibers; the second one, named “Hydrodynamic theory,” attributes sensitivity to the stimulation of the nervous endings due to the movement of the dentinal fluid, which is present in the dentinal tubes; and the third, named “Odontoblast theory,” in which the odontoblast (OD) itself is proposed as a receptor and signal transducer cell [1, 2]. Each of these theories has its flaws. On the one hand, although the most widely accepted is the hydrodynamic theory, the sensitivity does not account for the variability in the dentinal response against different natured stimuli. On the other hand, regarding the nerve theory, the sensitivity is difficult to explain because the penetration of the nerve endings seems to be limited to the deep dentin, which would render the surfaces that could be exposed to stimuli as innervated, which are the surface of the dentin and, eventually, the middle section of it. Regarding the viability of the odontoblast theory, recent findings allow us to glimpse the possibility that odontoblasts may, in fact, be sensory cells capable of triggering intracellular molecular responses to these external stimuli. This theory appears to be supported by recent studies that have shown the presence of transient receptor potential cation channel (TRP) membrane receptors in ODs, which in turn, have been localized in rat, mice, and human ODs using *in vitro* experiments, and they seem to be related to this nociceptive response [2–8].

The transient receptor potential vanilloid 1 (TRPV1) is characterized by its polymodal activation against chemical, thermal, mechanical, and pH-variation stimuli in neuronal cultures from human trigeminal ganglion (GT) [9], in rat and mouse dental primary afferent neurons [5, 10]. Regarding chemical stimulation, the most studied vanilloid agonist for chemical stimulation is capsaicin (CAP), which has a vanilloid functional group [3–8]. Additionally, it has been used to describe a desensitization process of the receptor, which would cause an analgesic effect [11]. Considering the structure-activity relationship, it is hypothesized that this receptor would also be activated with other molecules with a vanilloid functional group, such as eugenol (EUG). EUG is a phenolic vanilloid compound widely used in dentistry, and it has been described as an activator of the TRPV1 receptor in HEK-293 transfected cells; however, its effect has not yet been studied in cells with an odontoblast phenotype [12].

Considering all of the above information, issues arise: first, the proposed theories might not precisely clarify the inherent molecular process underlying pathology, as there seems to be a gap of knowledge regarding this topic [1, 2]. Second, various therapeutic strategies have been developed, such as the application of topical fluoride, “desensitizing” toothpastes, gels, varnishes, or composite resin restorations. However, due to their high therapeutic failure frequency, these alternatives have not been able to solve the problem [1]. Finally, there is no availability of a functional analysis of the polymodal activation of the TRPV1 receptor in human odontoblast like-cells (hOLCs) and, specifically, using EUG―in the case of chemical stimulation― [12] all of which motivated the present investigation.

Consequently, the proposed aim was to determine the expression, location, and functional activity of the pain receptor TRPV1 in an *in vitro* model of hOLCs by subjecting them to chemical stimuli with CAP and EUG, thermal stimuli, and exposure to several pHs. We aimed to propose a model for the study of pulp pain, providing valuable information that can be used to develop a therapeutic alternative for the treatment of dentinal hypersensitivity.

## 2. Materials and Methods

### 2.1. Obtaining Human Dental Pulp Stem Cells (hDPSCs)

Following the protocol proposed by Baldion et al. 2018 [13], healthy human third molars, free of caries and restorations, with the indicated extraction of patients between 14 and 18 years of age, with prior informed consent and endorsement of the Institutional Ethics Committee (B.CIEFO-122-18) were obtained. The teeth were decontaminated, and the pulp was extracted and immersed in Dulbecco's Modified Eagle Culture Medium (DMEM) low in glucose (Hyclone, Thermo Scientific, Bremen, Germany), supplemented with fetal bovine serum (FBS) (Gibco; Thermo Fisher Scientific) at 10% and antibiotics, in a dissociation medium with collagenase (3 mg/ml) and dispase (4 mg/ml) (Sigma-Aldrich; St Louis, MO, USA) for 16 h in an incubator with a humidified atmosphere with 5% carbon dioxide (CO_2_) at 37°C. The cell suspension was centrifuged, and the pellet was resuspended to be seeded in 25 cm^2^ flasks, making subcultures until reaching 70% confluence. To evaluate the behavior of cell proliferation, the population doubling time (PDT) was calculated by counting with a hemocytometer using trypan blue (Gibco, Thermo Fisher Scientific) applying the following formula: (*t*_2_−*t*_1_)/3.32 × (Log *n*_2_−Log *n*_1_) where *t* corresponds to the days in culture and *n* the number of cells, the result was verified using the Doubling Time software [14].

The hDPSCs were characterized using flow cytometry according to the criteria of the International Society for Cell Therapy [15], with the use of the FACSCalibur cytometer (BD Biosciences; San Jose, CA, USA) and a phenotyping cocktail (Miltenyi Biotec; Bergisch Gladbach, Germany) that included antibodies to detect the markers CD73, CD90, CD105, CD34, CD14, CD20, and CD45. In order to corroborate the undifferentiated mesenchymal phenotype and its potential for differentiation toward different lineages. Differentiation toward the osteoblastic, chondroblastic, and adipoblastic phenotype was induced for 21 days. The StemPro® Osteogenesis Differentiation Kit (Gibco, Thermo Fisher Scientific) differentiation medium was used to obtain the osteoblast culture, which was stained with alizarin red (Sigma-Aldrich) in order to evaluate calcification nodules and mineralized matrix formation. The StemPro® Chondrogenesis differentiation kit (Gibco, Thermo Fisher Scientific) was used to obtain chondrocytes, and the Alcian Blue stain (Sigma-Aldrich) was performed in order to identify their ability to proteoglycans synthesize. Finally, to obtain adipocytes, the differentiation medium StemPro® Adipogenesis differentiation kit (Gibco, Thermo Fisher Scientific) was used, and an Oil RedO stain (Sigma-Aldrich) was carried out in order to identify neutral lipids. Images were obtained with the Leica DM2500 Microscope (Leica Microsystems; Wetzlar, Germany) [16].

### 2.2. Differentiation Process of hDPSCs to hOLCs

A DMEM medium was used, supplemented with FBS, 100 U/ml penicillin, and 100 ug/ml streptomycin; 0.1 *μ*M dexamethasone (Sigma-Aldrich), 5 mM *β*-glycerophosphate (Santa Cruz, CA, USA), 50 *μ*g/mL ascorbic acid (Sigma-Aldrich), and 10 ng/mL TGF-*β*1 (Abcam, Cambridge, MA, USA). The hDPSCs were exposed to the differentiation medium for 7, 14, and 21 days in an incubator at 37°C in a humidified atmosphere containing 5% CO_2_ [13], and the behavior of the TRPV1 receptor expression within the differentiation process was determined.

An indirect immunofluorescence technique was performed for the odontogenic markers, dentinal matrix protein-1 (DMP-1), and dentinal sialoprotein (DSP) following the protocol reported by Baldion et al. [13]. Briefly, the cells (8 × 10^3^ cells/well) were seeded on poly-L-lysine-treated glass coverslips, which were fixed with 4% paraformaldehyde (PFA), and permeabilized with Triton X-100, to subsequently block with 10% goat serum. Cells were incubated at 37°C with anti-DMP-1 human polyclonal primary antibody produced in rabbit (Sigma-Aldrich), diluted 1 : 50 in blocking serum. The samples were then incubated with goat Fluorescein isothiocyanate (FITC) conjugated anti-rabbit IgG secondary antibody (Thermo Fisher Scientific) diluted 1 : 200 in PBS at room temperature. The nuclei were counterstained with Hoechst, and the slides were mounted with VectaShield (Vector Laboratories, Inc. Burlingame, CA, USA) for observation under the Zeiss Axio Imager A2 microscope (Göttingen, Germany) with the AxioVision software. The presence or absence of the protein in cells and its location were evaluated in three independent experiments (*n* = 3). In the same way, the procedure for the DSP marker was carried out using the primary polyclonal human antidentin sialophosphoprotein (DSPP) antibody produced in rabbit (Abcam, Cambridge, MA, USA), which is specific for the N-terminal portion that corresponds to natural cleavage by DSP [13].

### 2.3. Detection of the TRPV1 Receptor in hOLCs

#### 2.3.1. TRPV1 Gene Expression

The polymerase chain reaction technique with real-time reverse transcriptase (RT-qPCR) using SYBR Green with the Luna® Universal One RT-qPCR Kit (New England BioLabs; USA) and the CFX96 Real-Time Thermal Cycler detection system (BioRad; Hercules, CA, USA) was used to determine TRPV1 gene expression. Amplification conditions were as follows: retrotranscription for 10 min at 55°C, initial denaturation for 1 min at 95°C, 40 cycles of amplification with an alignment temperature of 58°C using the following primers (Macrogen®) TRPV1: Forward 5′-GGCTGTCTTCATCATCCTGCT GCT-3′ Reverse 3′ GTTCTTGCTTCTCTGTGCGATCTTGT-5′ (NM_080706 : 118 bp) and *β*-actin was used as a housekeeping gene: Forward 5′-CGCCGCCAGCTCACCATG-3′ Reverse 3′-CACGATGGAGGGGAAGACGG-5′ (NM_000576.3 : 120 bp). The PCR efficiency was calculated using LinRegPCR (Academic Medical Center, AMC, Amsterdam, Netherlands), and the relative quantification of the amplification was performed with Schefe's method [13, 17].

For the TRPV1 identification and localization, immunocytochemical and immunohistochemical assays were performed. For this, 8 × 10^3^ cells/well were seeded on poly-L-lysine-treated coverslips in a 24-well plate. After reaching 30% confluence, they were fixed with 4% PFA, permeabilized, and incubated with a rabbit primary polyclonal antibody, specific for human TRPV1 (Thermo Fisher Scientific) in blocking serum. Alexa Fluor 594 coupled to streptavidin (Thermo Fisher Scientific) was used for detection. The nuclei were counterstained with Hoechst and were observed in the A2Axio Imager A2 microscope (Zeiss, Göttingen Germany) and analyzed with the AxioVision software. The presence or absence of the protein in cells and its localization were evaluated in three independent experiments (*n* = 3) [13].

### 2.4. TRPV1 Functional Evaluation

#### 2.4.1. TRPV1 Receptor Agonists

Both EUG and CAP (Sigma-Aldrich) were dissolved in dimethylsulfoxide (DMSO) to obtain a stock solution and kept refrigerated at −20°C. The final concentration of DMSO was less than 0.1% (v/v) in order not to affect the membrane currents action potential and the intracellular Ca^2+^ ([Ca^2+^]i) concentration [8]. The cell viability was evaluated using the resazurin technique to establish the half-maximal effective concentration (EC50) of both substances. 25 × 10^3^ cells/well were cultured in 96-well plates for 20 h. Cells were treated for 1 h with 0.1; 0.5; 1; 5 and 10 mM EUG; after the time had elapsed, it was removed, and 4.4 *μ*M resazurin (Sigma-Aldrich) at 10% v/v was added to each well, starting from an initial solution of 44 *μ*M, and incubated for 1 h in the incubator at 37°C in a humidified atmosphere containing 5% CO_2_. Once the incubation time had elapsed, the plates were read in the Infinite M200 spectrofluorometer (Tecan; Männedorf, Switzerland) at a wavelength of 494/520 nm (excitation/emission). With the fluorescence intensity measurement, the reduction percentage of resazurin to resorufin of the treated groups concerning the control of untreated cells was calculated, taken as 100%. Three independent experiments were analyzed, with six replicates each one (*n* = 18). In the same way, cells were exposed to 0.5; 1; 5; 10 and 50 *µ*M CAP for 1 h following the same protocol. With the data obtained, the EC50 was calculated by performing the concentration-response curves for each compound using the GraphPad Prism 7.0 software (GraphPad Software, San Diego, CA, USA).

#### 2.4.2. Polymodal Stimuli Assays

Polymodal stimulation assays were performed measuring the [Ca^2+^]i change using the Fluo4-AM Kit (F14201 Invitrogen, Thermo Fischer Scientific) as criteria to determine the activation of TRPV1 in hOLCs. For this, 25 × 10^3^ cells/well were seeded in 96-well plates in DMEM medium supplemented with 10% FBS, 100 IU/ml penicillin, 100 *µ*g/ml streptomycin, and 10 ng/ml TGF-*β*1. Cells were loaded with 4 *µ*M Fluo4-AM at 37°C for 60 min in the dark. After time elapsed, the Fluo4-AM was removed, and 1x PBS was added for 30 min. Subsequently, the PBS was removed, and each test substance was added. The reading was done in the spectrofluorometer (TECAN, Infinite M200) after 1 min and 20 min of exposure at 494/520 nm (excitation/emission). The difference between the fluorescence obtained and the baseline of unstimulated cells was used as an indirect measure of the changes in [Ca^2+^]i. Each experiment was carried out in triplicate with 3 replicates each one (*n* = 9).

For the chemical stimulation test with EUG and CAP, the calculated EC50 was used. For the thermal stimulation assay, cells were subjected to DMEM medium without phenol red in a range of 37 to 49°C. For the stimulation with different pHs assay, the basal DMEM medium (pH 7.4) was progressively added 1 N hydrochloric acid (HCl) to acidify it and obtain the pH of 4, 5, and 6. 5 N sodium hydroxide (NaOH) was added to obtain pH of 8, 9, 10, 11, and 12. The pH was measured using a potentiometer (Hanna Instruments HI 2210, Rhode Island, USA).

### 2.5. Data Analysis

A spreadsheet of the Excel program (Microsoft Office 2010) was constructed, and the statistical package SPSS Version 23 was used. For the variables with normal distribution, the mean and standard deviation (SD) were used. Qualitative variables were described in terms of frequencies and proportions. A one-way ANOVA analysis of variance with a Tukey post hoc test was used using an alpha value < 0.05.

## 3. Results

### 3.1. Obtaining hDPSCs and the Differentiation Process of hOLCs

**Figure 1:**
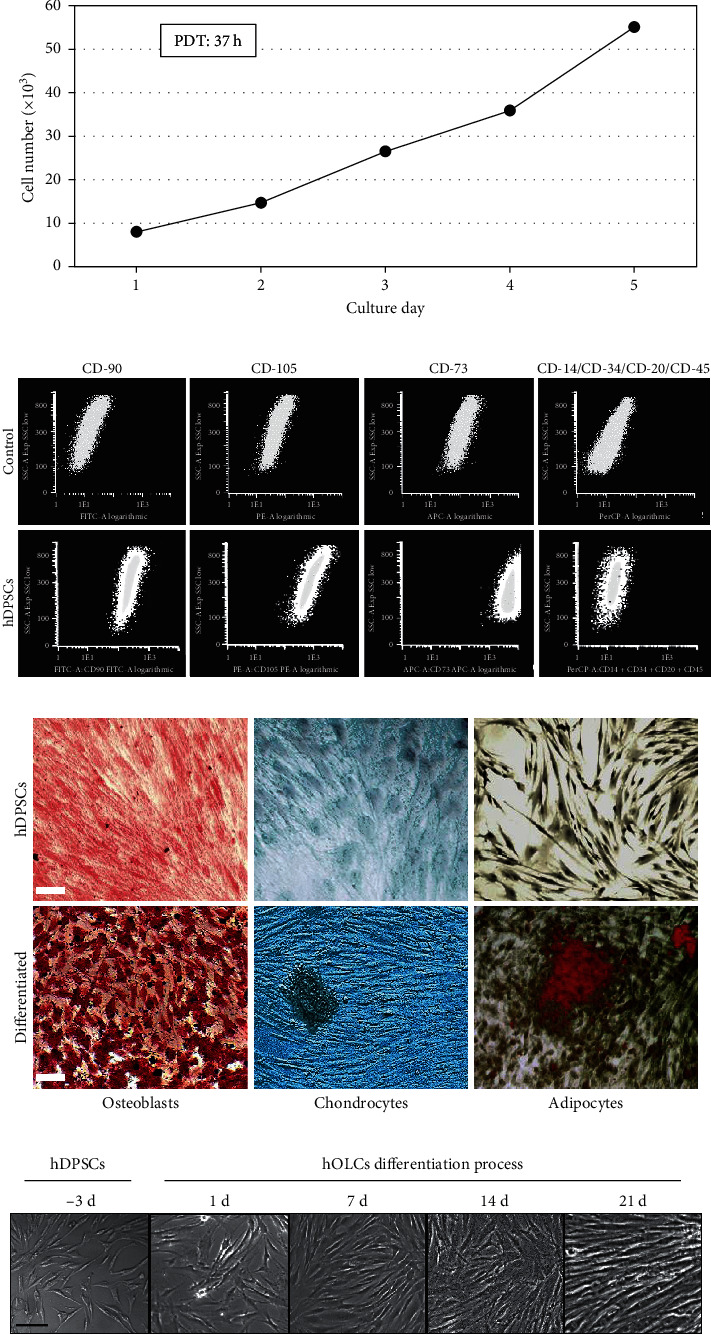
Phenotypic characterization of hDPSCs and morphological cell appearance during the cell differentiation process. (a) The PDT was calculated considering the cell count performed in the first 96 hours (4 days) of cell culture, using a hemocytometer. Results are shown as the average of three independent experiments in triplicate (*n* = 9). (b) The undifferentiated mesenchymal phenotype was verified using flow cytometry by using the cocktail for phenotyping of Miltenyi, which demonstrated positivity for mesenchymal markers CD90, CD105, and CD73 and negativity for the early hematopoietic markers CD14, CD20, CD34, and CD45. A total of 100,000 events were acquired with the use of the FACSCalibur cytometer, and the data were analyzed using FCS Express software. (c) To evaluate the differentiation potential of hDPSCs, they were exposed to differentiation media for the three lineages for 21 days and the respective stains were performed in order to corroborate the acquired phenotype. The osteoblasts were stained with alizarin red, and the formation of mineralization nodules is evidenced. The chondrocytes were stained with Alcian blue, positive labeling of proteoglycans is evidenced, and adipocytes were stained with OilO red; a positive staining of fatty acids is shown. Scale bar: 100 *μ*m. (d) Phase contrast microscopy images showing hDPSCs with tapered, fibroblastic morphology, as well as hOLCs cells during the differentiation process at 7, 14, and 21 days. A trend toward organized training in parallel lines is shown. Leica DM2500 Microscope (Leica Microsystems; Wetzlar, Germany) was used. Scale bar: 100 *μ*m.

hDPSCs showed an adequate monolayer adherence to the plastic, with a spindle-like shape and a fibroblast-like morphology, having a good cell proliferation rate with a PDT of 37 h (Figure 1(a)). They were characterized using flow cytometry to evaluate the expression of specific markers of undifferentiated stem cells. Its mesenchymal phenotype was demonstrated by the positivity for CD90, CD105, and CD73 in more than 95% of the cells and negativity for the early hematopoietic markers CD14, CD20, CD34, and CD45 (Figure 1(b)) in less than 1% of cells and an *in vitro* differentiation capacity toward several lineages, such as osteoblasts, chondrocytes, and adipocytes (Figure 1(c)).

Subsequently, hDPSCs were differentiated for 7, 14, and 21 days in an odontogenic induction medium to obtain hOLCs. There were no apparent morphological changes during the differentiation process, and an optimal proliferation was maintained (Figure 1(d)).

**Figure 2:**
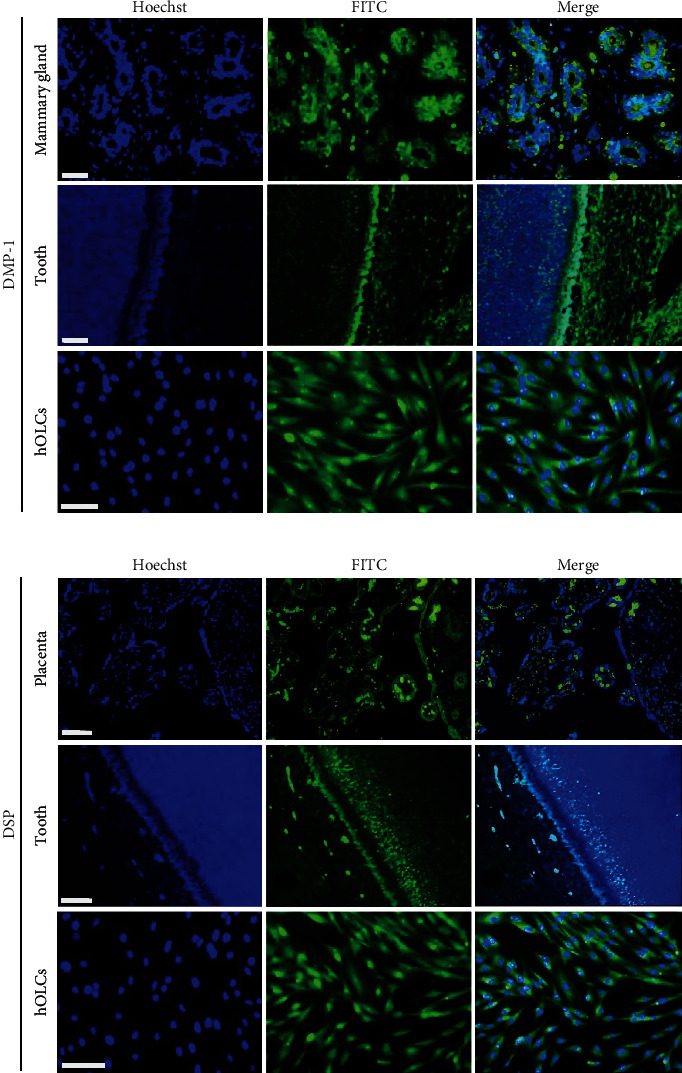
Immunocytochemical detection of odontogenic markers DMP-1 and DSP in hOLCs. Positive immunostaining (green) of tissue control (a) mammary gland for DMP-1 and (b) placenta for DSP. Positive staining of dental tissue in the odontoblast layer and mineralization in front of the dentin. To determine the presence and location of the odontogenic markers in the differentiated cells, an anti-DMP-1 (a) or anti-DSP (b) antibody conjugated with FITC (green) was used, and the nuclei were counterstained with Hoechst stain (blue). Note the positivity of these markers in hOLCs, with both a nuclear and cytoplasmic distribution. A Zeiss Axio Imager A2 microscope was used to obtain the images. Scale bar: 50 *µ*m.

Additionally, using immunofluorescence, the odontoblast phenotype of hOLCs was verified at 21 days of differentiation, and the odontogenic markers DMP-1 and DSP were identified. Dental tissue and mammary gland or placenta were used as controls, respectively (Figure 2).

### 3.2. Detection of the TRPV1 Receptor in hOLCs

#### 3.2.1. Evaluation of TRPV1 Receptor mRNA and Determination of Its Presence in hOLCs

**Figure 3:**
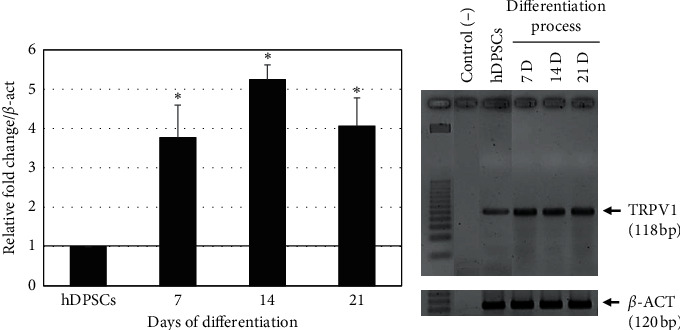
Relative quantification of TRPV1 at different differentiation times using RT-qPCR. (a) Relative quantification of TRPV1 transcripts using RT-qPCR in hOLCs cells at 7, 14, and 21 days of differentiation, compared to hDPSCs, which were used as baseline, and considered as 1. An overexpression of transcripts was observed for all the differentiation times. Results are presented as mean ± standard deviation (SD) of three independent experiments in triplicate (*n* = 9). The asterisks show statistically significant differences between hDPSCs cells and the control (*p* < 0.05). (b) Agarose gel electrophoresis (2%) of the TRPV1 products of RT-qPCR. An increase in the expression of the transcripts is observed at 7, 14, and 21 days of differentiation, compared to hDPSCs. *β*-actin (*β*-ACT) was used as a housekeeping gene. GelDoc Go System reader was used (BioRad, Hercules, CA, USA).

RT-qPCR revealed a differential pattern of spatiotemporal expression of the TRPV1 transcripts at 7, 14, and 21 days of differentiation (*p* < 0.05) (Figure 3(a)) regarding *β*-actin (*β*-ACT). Additionally, agarose gel electrophoresis of the products was performed, and a higher expression of TRPV1 mRNA was observed in differentiated hOLCs at 7, 14, and 21 days, compared to hDPSCs (Figure 3(b)).

**Figure 4:**
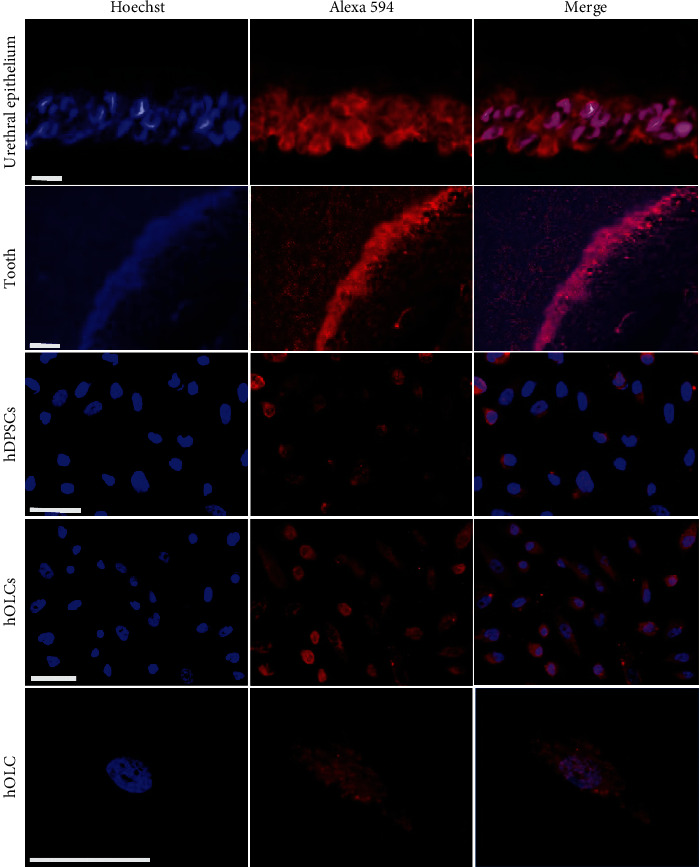
Immunocytochemical detection of TRPV1 receptor in hOLCs. Immunocytochemical identification of TRPV1 in the control of urethral epithelial tissue, in dental tissue, in hDPSCs cells, and in hOLCs. Anti-TRPV1 antibody conjugated with Alexa fluor 594 (red) was used to obtain the protein labeling and the nuclei were counterstained with Hoechst stain (blue). Note the positive staining in the dental tissue located in the area of the odontoblast palisade. Additionally, in hOLCs cells, an increase in this immunostaining localized in the membrane is evident, compared to hDPSCs. Zeiss Axio Imager A2 microscope was used. Scale bar: 50 *µ*m.

The presence and location of the TRPV1 receptor were determined using immunofluorescence (Figure 4).

### 3.3. TRPV1 Receptor Functional Evaluation

**Figure 5:**
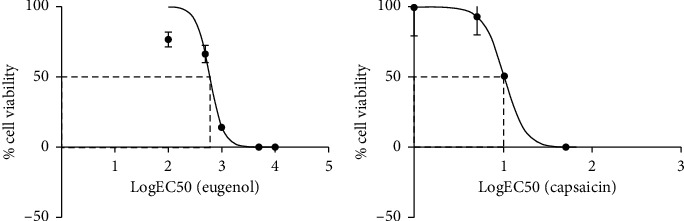
Calculation of EUG and CAP EC50 in hOLCs using resazurin technique. The EC50 was calculated from the resazurin assay in hOLCs exposed to different concentrations of the two compounds. (a) EUG EC50. Concentration range: 10, 5, 1, 0.5, and 0.1 mM. LogEC50 : 2.781, equivalent to 603.5 *μ*M. (b) CAP EC50. Concentration range: 50, 10, 5, 1, and 0.5 mM. LogEC50 : 1.004, equivalent to 10.1 *μ*M. GraphPad Prism 7.00 software was used. The curves represent the results of 3 independent experiments with six replicates each (*n* = 18).

Cell viability was determined using the resazurin technique, and the EC50 was found for 603.5 *µ*M EUG (Figure 5(a)) and 10.1 *µ*M CAP (Figure 5(b)).

### 3.4. Polymodal Stimuli Assays

The cells were stimulated for 1 min (simulation of immediate exposure) and for 20 min (simulation of chronic exposure). After stimulation, the measurement of relative fluorescence units (RFU) was performed using spectrofluorimetry (TECAN, Infinite M200). Means ± SD of three independent experiments with three replicates each (*n* = 9) are shown. Analysis of variance one-way ANOVA and Tukey's post hoc analysis established a statistically significant difference with a *p* < 0.05.

#### 3.4.1. Chemical Stimulation

**Figure 6:**
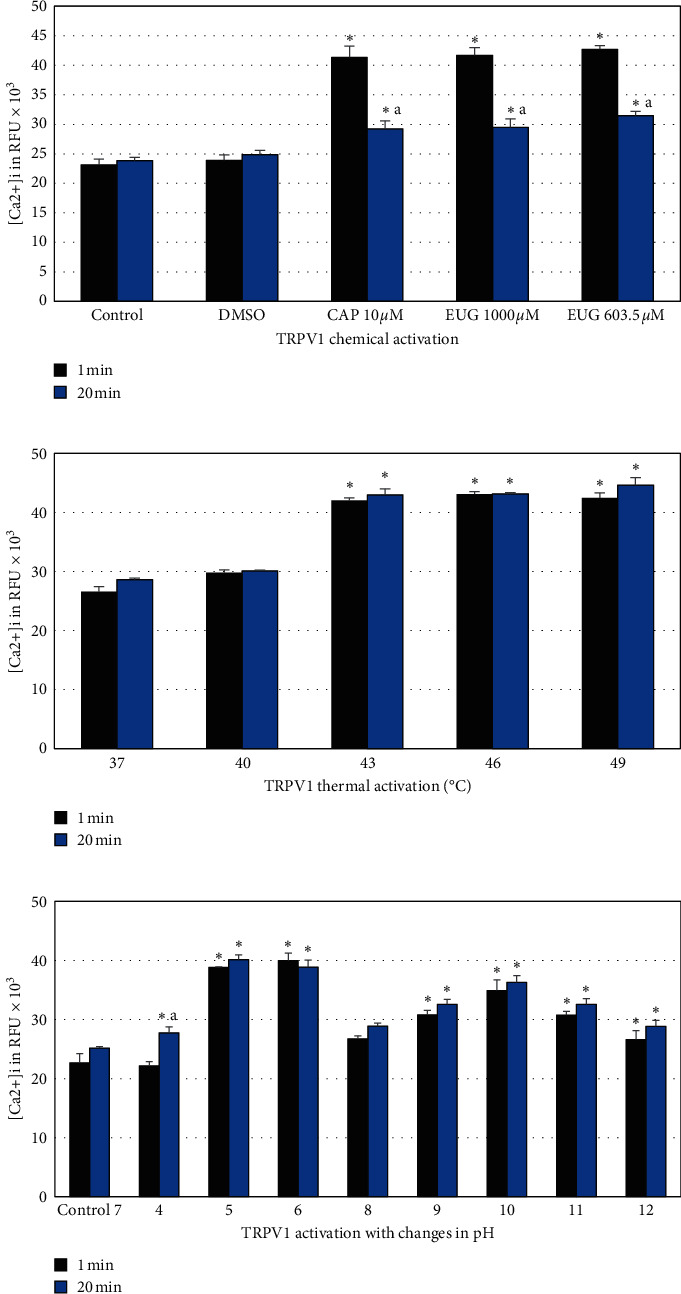
Chemical, thermal, and pH stimuli of TRPV1 receptor in hOLCs. In the polymodal stimulation assays, 1 and 20 min of exposure to the stimulus were evaluated using the Fluo 4 AM probe. (a) Chemical stimulation of the TRPV1 receptor. Statistically significant differences were found between the test groups at the different exposure times evaluated (1 and 20 min) with both CAP and EUG. TRPV1 receptor desensitization was confirmed at 20 min with both vanilloid substances, and no differences from the control were identified. (b) Thermal stimulation activation at 43°C. No statistically significant differences were found between the test groups at the different exposure times evaluated (1 and 20 min). (c) Stimulation with changes in pH. Activation was marked at pH 5 and 6. Relative fluorescence units (RFU) were detected at a wavelength of 494/520 nm (excitation/emission) using a TECAN microplate reader. Results are shown as the mean ± SD of three independent experiments in triplicate (*n* = 9). Each group was compared to its respective control, and the asterisks show statistically significant differences between them (*p* < 0.05). The lowercase letter (a) shows statistically significant differences between the test groups at the different times evaluated (1 and 20 min) (*p* < 0.05).

The hOLCs were stimulated with CAP (10 *µ*M) and EUG (603.5 and 1000 *µ*M), and unstimulated cells were used as a control. Additionally, vehicle dimethyl sulfoxide (DMSO) < 1% was evaluated. The results showed that the cells exposed to CAP and EUG had a significant RFU increase, indicating a TRPV1 receptor activation, compared to the control and vehicle (DMSO) for the two evaluated times (1 and 20 min). Assuming that the stimulation for 20 min is equivalent to chronic exposure to the compound, it was evident that there was a marked difference between these measurements compared to the immediate exposure (1 min), which demonstrated a marked decrease in receptor activation (Figure 6(a)), related to receptor desensitization.

#### 3.4.2. Thermal Stimulation

In hOLCs stimulated with temperatures ≥43°C, an increase in [Ca^2+^]i and, consequently, in TRPV1 receptor activation, was observed compared to 37°C and 40°C. There was no difference between the measurements at 1 and 20 min (Figure 6(b)).

#### 3.4.3. Stimulation with Different pHs

For the physiological pH of 7, used as a reference point, a consistent increase in RFU was observed, which is related to the TRPV1 receptor activation from a pH of 5 onwards. This activation was more noticeable, specifically at pH 5 and 6, both in immediate exposure (1 min) and chronic exposure (20 min). Additionally, the only group in which a difference was observed between the exposure times (1 and 20 min) was the one stimulated with a pH of 4 (Figure 6(c)).

## 4. Discussion

This study demonstrated the presence, localization, and polymodal activation of the TRPV1 receptor in hOLCs. First, the phenotypic characterization of both hDPSCs and hOLCs obtained after odontogenic differentiation could be performed, which is a useful tool for the study of dental sensitivity. Second, using RT-qPCR, it was possible to demonstrate not only the expression of the TRPV1 receptor in these hOLCs, but also an increase in the expression of TRPV1 transcripts at 7, 14, and 21 days of differentiation in hOLCs, compared to hDPSCs cells (*p* < 0.05). The highest expression at 14 days of differentiation could be related to the fact that, at this stage, cells are characterized as mature secretory odontoblasts. Therefore, it becomes a more specialized sensitive cell that requires a more developed sensory function. This suggests that hOLCs could be considered sensitive cells and that such a condition could be associated with the expression of the TRPV1 receptor membrane. Similar results were found using immunocytochemistry. First, in dental tissue, TRPV1 is specifically located in the area of the odontoblast palisade. Secondly, TRPV1 presented a membrane distribution with higher immunostaining in hOLCs than in hDPSCs. Together, these two findings confirm the relationship between the presence of the TRPV1 receptor and the obtention of the odontoblast phenotype [8, 18].

TRPV1 receptor activation was carried out under three conditions: chemical stimulation, thermal stimulation, and exposure to different pHs. Regarding chemical stimulation, it was possible to observe a difference in the concentration necessary to activate the TRPV1 receptor. According to the cell type, it was found that the EC50 calculated for EUG was 603.5 *μ*M in hOLCs, while the EC50 reported in HEK-293 cells and trigeminal neurons was 1 mM [12]. This evidence allows us to appreciate a difference in the sensitivity to chemical stimulation performed with EUG as an agonist in OD and in other cell models.

In addition, it was possible to observe the TRPV1 receptor activation with CAP at 10 *µ*M, and with EUG at 1000 and 603.5 *µ*M, with very similar behaviors. The activation of TRPV1 with CAP in different cell types, which has been previously reported [3–8], is based on the attribution of such activation to the specific binding of CAP with Glu-571 and Thr-551 residues of the receptor, which causes a conformational change that, in turn, induces the opening of the channel. Some authors hypothesize that this mechanism of chemical receptor activation could be applicable to the entire group of capsaicinoids, including EUG [19].

Second, regarding thermal stimulation, TRPV1 activation was observed in hOLCs at temperatures ≥43°C, a result consistent with that reported in previous studies carried out in human OD [8], mouse OD [4], and rat primary afferent neurons [5]. Previous studies have attributed this thermal activation to two potential complementary mechanisms. On the one hand, it is proposed that the protein kinase A/A-kinase anchoring proteins (PKA/AKAP) signaling pathway is involved in the thermal hyperalgesia mechanism through the phosphorylation of a Ser-502 residue located between the TRPV1 S2-S3 linker, which would promote an ionic pore opening [20]; on the other hand, it is suggested that this ionic pore could act as a thermal sensor [21]. However, the lack of clarity regarding the molecular details of TRPV1 activation by increasing the temperature has led to the suggestion of a third mechanism, according to which the ankyrin repeat domain (ARD) could undergo structural changes in response to heat, which could determine the TRPV1 activation [22].

Instead, in terms of exposure to different pH values, a significant activation of the TRPV1 receptor in hOLCs subjected to acidic pH (5 and 6), both in the immediate (1 min) and chronic (20 min) exposure, was identified. This is in agreement with the findings of a previous report, which registered a TRPV1 receptor activation at a pH lower than 6 [6]. The results demonstrated a discrepancy between the two exposure times (1 and 20 min) at pH 4, with a significant activation with a sustained exposure for 20 min. These findings are consistent with the functional study by Tsumura et al. on rat OD, in which receptor activation was demonstrated after 2 min of exposure using an acidic solution at the same pH, an activation that was conserved in subsequent measurements [6].

Additionally, receptor activation was observed at basic pHs―between 9 and 12―an unprecedented report on previous studies in OD. Within this range, the most significant activation occurred at pH 10, although it was not as remarkable as in the case of acidic pHs [6]. Previous investigations carried out in mouse GT neurons and in transfected HEK-293 cells demonstrated the activation of TRPV1 by exposure to ammonium (NH_4_^+^) and by intracellular alkalinization, which causes the deprotonation of the Hys-378 residue in the cytoplasmic domain of the receptor. This, in turn, would cause new intra- and intermolecular interactions, leading to a conformational change that would translate into receptor activation. Such activation occurred at a pH > 8 and at a pH of 7.8–9.5 in the first and second systems, respectively, which is consistent with the findings of our study [23].

At this point, the activation of the TRPV1 receptor in hOLCs is taken for granted at the three conditions described, namely chemical stimulation, thermal stimulation, and exposure to different pH values. Next, it is important to raise the hypotheses that have tried to establish the possible mechanism by which such activation translates into pain and its role in dental nociception.

First, it has been proposed a possible “neuro-odontoblast” interaction due to TRPV1 receptor activation, caused by thermal or mechanical stimulation, due to the entry of [Ca^2+^]i into the OD, which can cause the depolarization of its membranes. In turn, the entry of Ca^2+^ and membrane depolarization would release stimulant “transmitters” that could activate the nearby nerve endings [3, 24].

Subsequently, it was possible to establish an association between the influx of Ca^2+^ involved in TRPV1 receptor activation and the stimulation of adenosine triphosphate (ATP) production in OD. This finding contributes to the elucidation of the communication mechanism between OD and nerve fibers thanks to the presence of purinergic-type receptors P2X (ionotropic) and P2Y (G protein-coupled), both in OD and nerve endings. ATP release mechanisms, namely Ca^2+−^ dependent mobilization of vesicles, and transportation of ATP via connexin 43 or pannexin 1 (PANX-1) as a membrane or hemichannel transporters, have been recorded [2, 25–27]. These findings offer strong support for the hypothesis of the neuro-odontoblast interaction and for the postulate of the sensory implications of ATP release.

Thus, the findings of the relationship between the TRPV1 receptor and nociceptive transmission suggest that research aimed at achieving analgesia could be devoted to the study of the desensitization of this receptor. Regarding receptor desensitization via chemical stimulation, such as that found using EUG in our study, several studies clarify and support the achievement of analgesia with the use of vanilloids [11].

It is possible to characterize desensitization caused by the chronic exposure to agonists (20 min), as a process remarkably dependent on the entry of Ca^2+^ and the activation of several intracellular signaling pathways due to the importance of Ca^2+^ as a second messenger. Previous evidence showed that in Chinese hamster ovary derived cells (CHO) transfected and stimulated with CAP, the increase in [Ca^2+^]i caused marked TRPV1 receptor desensitization, induced by the intracellular dephosphorylation of Ser-116 residue. It has been proposed that the vanilloid substance would be responsible for inactivating PKA, thus leading to a TRPV1 receptor desensitized state [28], similar to that obtained by the action of calcineurin [29, 30].

A desensitization mechanism in rat dorsal root ganglion neurons (DRG) has also been proposed for activating calcineurin, which would lead to the dephosphorylation of residues Ser-502 and Thr-704 and, therefore, to TRPV1 desensitization which would lead to the activation of the enzyme Ca^2+^/calmodulin kinase II (CaMKII), which would rephosphorylate the receptor at residues Ser-502 and Thr-704. This process of rephosphorylation returns the TRPV1 receptor to its activation capacity, provided that other important residues, such as those mentioned above, are previously phosphorylated by PKA or protein kinase C (PKC). The authors highlight this balance between phosphorylation and dephosphorylation, which could be more complex and involve more enzymatic signaling pathways [31].

Additionally, it has been demonstrated a desensitization mechanism mediated by calmodulin binding to a 35-amino-acid segment in the C-terminal portion of TRPV1 in HEK-293 cells [32]. In addition, a subsequent study reported a calmodulin binding domain located in the N-terminal portion of TRPV1, which was made up of a 33-amino-acid section adjacent to the ARD [33]. Likewise, the importance of this ARD section has been previously mentioned [33], insofar as an ATP is attached to it as a “sensitizer” that, in conjunction with phosphatidylinositol 4,5-bisphosphate (PIP2), is ready to be activated. After the activation of TRPV1, the influx of [Ca^2+^]i would cause the release of ATP and the degradation of PIP2 via calmodulin, which leads to receptor desensitization [34, 35].

Finally, another study carried out in rat DRG neurons and in HEK-293 cells demonstrated that a chronic exposure (20 min) to CAP (100 nm and 1 *μ*M, respectively) promotes the internalization and degradation of the receptor in a manner that depends on dose, time, and Ca^2+^ concentration. The aforementioned internalization by the endocytic pathway is modulated by PKA [36]. In addition, it has been found that PKA actively participates in the functional insertion of TRPV1 in the plasma membrane; therefore, its inactivation, caused by CAP, would induce a reduction in membranal TRPV1 as an additional mechanism. Thus, the available evidence indicates that chronic exposure to vanilloids could be used to modulate the membrane expression of TRPV1 [36].

In summary, multiple mechanisms of chemical desensitization with vanilloids have been reported, namely, TRPV1 dephosphorylation by calcineurin, calmodulin-dependent kinases, inactivation of PKA [28–30], calmodulin binding [32, 33], ATP release or depletion of PIP2 from the plasma membrane [34, 35], and internalization and degradation of the membrane receptor TRPV1 [36]. It should be noted that all the aforementioned mechanisms show significant potential to achieve analgesia. As has been recognized throughout this study, such mechanisms have been studied mainly in neuronal cultures of DRG and GT of rats and mice, in cultures of transfected HEK-293 cells, and in cultures of transfected CHO cells. However, to date, no study investigating these desensitization mechanisms in OD has been reported.

Consequently, regarding desensitization in OD, our study was able to establish that a higher concentration of EUG was required to achieve the same effect induced by CAP after a 20-min exposure (10.1 *µ*M CAP and 603.5 *µ*M EUG). Some authors have proposed that the response patterns of EUG are distinct from those of CAP. They suggested that TRPV1-independent mechanisms also appear to be involved in the pharmacological effects of EUG [12]. Unlike CAP, EUG has been widely used in dentistry and its efficacy for relieving toothache has been extensively demonstrated due to its antioxidant effect [37], its antinociceptive effect (related to the inhibition of voltage-gated sodium and calcium channel currents) [38, 39], its anti-inflammatory effect (related to significant inhibition of PGE2 production and to a suppression of the cyclooxygenase-2 (COX-2) gene expression) [40], and its antimicrobial effect (related to its broad-spectrum action against Gram-positive and Gram-negative bacteria, fungi, and virus) [41].

Regarding receptor desensitization by thermal stimulation, in this study, no evidence of desensitization induced by this type of stimulus was found, with no variation between the measurements at 1 min and after 20 min of exposure. These results are consistent with the absence of reports of thermal stimulation-induced receptor desensitization in the available literature. Regarding the receptor desensitization by exposure to different pH levels, no evidence in favor of it was found in the present study. An earlier study obtained TRPV1 receptor desensitization in a single instance through exposure to an acidic solution at pH 5.5. Furthermore, it was observed that, unlike the stimulation induced by vanilloids, desensitization would occur by a mechanism independent of CaMKII, which suggests that the process involves other factors and signaling pathways [31].

Finally, the clinical importance of the evidence found in the present study lies in that, although EUG has fallen into disuse in dentistry because of its interference with composite resin polymerization, it has potential in the chemical activation and consequent desensitization of TRPV1 receptor. This projects it as a therapeutic alternative for dentinal hypersensitivity. This possibility is, of course, mediated by future research, which may support the clinical use of this vanilloid.

Additionally, to the thermal activation of the TRPV1 receptor, it must be considered that the pulp-dentinal complex is permanently exposed to high temperatures, typical of the consumption of hot foods or drinks. Additionally, in clinical contexts, such thermal activation can be caused by dental protocols or the use of dental materials with exothermic reactions. It should be remembered that all these procedures could activate TRPV1 receptors, which could cause pain or postoperatory sensitivity.

Furthermore, some dental materials used in clinical practice involve the exposure of the pulp-dentinal complex to varied pHs—basic ones, such as calcium hydroxide, or acidic ones, such as orthophosphoric acid, polyacrylic acid, some self-etching adhesives, or zinc phosphate cement, among others. For this reason, the study of the activation mechanisms of TRPV1, or other receptors of this family, associated with the exposure to different pH values, is necessary to improve clinical practice and to possibly relieve sensitivity after certain dental procedures.

In summary, the present study demonstrated the presence, membrane localization, and the TRPV1 receptor polymodal activation in a hOLC model. These findings are important because they are considered as the initial step for the construction of a study model of pulp pain, which represents a contribution to the development of therapeutic alternatives for dentinal hypersensitivity based on EUG as a desensitizing agent.

## 5. Conclusions

Within the aims proposed in this study, it was possible to demonstrate, based on evidence, the expression and localization of the TRPV1 receptor in a hOLC model, which represents a contribution of singular relevance to understanding the complexity of dentinal sensitivity. First, the present study demonstrated that the TRPV1 receptor is activated in response to a wide spectrum of conditions, namely, chemical stimulation, thermal stimulation, and exposure to different pH levels. Taken together, these results indicate that TRPV1 functions as an integrator for a variety of sensory inputs. Second, the present study made it possible to establish that the TRPV1 receptor exhibits a desensitization capacity under stimulation with the vanilloid agonist EUG, similar to that reported for the use of CAP. These findings represent a significant contribution to the study of analgesia and the control of dentinal hypersensitivity and are projected as the basis for the development of a therapeutic alternative for this pathology. Finally, it should be remembered that dentinal hypersensitivity is not only a frequent reason for consultation in dental practice but also that, at present, procedures aimed at treating it show a high therapeutic failure rate.

## Data Availability

The data used to support the findings of this study are available from the corresponding author upon request.
